# Fecal Microbiota Transplantation in Patients with Alcohol-Associated Cirrhosis: A Clinical Trial

**DOI:** 10.3390/jcm14175981

**Published:** 2025-08-24

**Authors:** Cristian Ichim, Adrian Boicean, Samuel Bogdan Todor, Paula Anderco, Victoria Bîrluțiu

**Affiliations:** Faculty of Medicine, “Lucian Blaga” University of Sibiu, 550024 Sibiu, Romania; cristian.ichim@ulbsibiu.ro (C.I.); paula.anderco@ulbsibiu.ro (P.A.); victoria.birlutiu@ulbsibiu.ro (V.B.)

**Keywords:** fecal microbiota transplantation, liver cirrhosis, hepatic encephalopathy, gut–liver axis, microbiota-based therapy

## Abstract

**Background**: Gut microbiota dysregulation is increasingly recognized as a key contributor to the progression of liver cirrhosis and its complications, particularly hepatic encephalopathy. Fecal microbiota transplantation (FMT) has emerged as a novel therapeutic strategy aimed at restoring intestinal microbial homeostasis and modulating systemic inflammation. **Methods**: This prospective, single-center clinical trial evaluated the short-term safety and efficacy of FMT in patients with alcohol-related liver cirrhosis. Clinical assessment, liver stiffness (via elastography), steatosis (controlled attenuation parameter), inflammatory biomarkers, and extended biochemical panels were analyzed at baseline, one week and one month post-FMT. A control group receiving standard medical therapy was used for comparison. **Results**: FMT was associated with a significant reduction in hepatic encephalopathy severity (*p* = 0.014), sustained improvements in liver stiffness (*p* = 0.027) and decreased steatosis (*p* = 0.025). At one month, C-reactive protein and neutrophil-to-lymphocyte ratio both declined significantly (*p* = 0.043), indicating a measurable anti-inflammatory effect. No serious adverse events were recorded. In comparison with controls, FMT recipients showed lower systemic inflammation and improved neuropsychiatric status. **Conclusions**: FMT demonstrated a favorable safety profile and yielded early clinical and biochemical benefits in patients with cirrhosis. These preliminary findings support the potential utility of microbiota-based interventions in chronic liver disease and warrant validation in larger, multicenter trials.

## 1. Introduction

The human gastrointestinal tract harbors a complex and diverse microbial ecosystem that plays essential roles in digestion, nutrient assimilation, epithelial development, and defense against pathogens [1,2]. Although the gut microbiome is typically individualized and relatively stable, its composition can be substantially influenced by environmental exposures, including diet, probiotics, prebiotics, viral infections, and especially antibiotic use [3,4].

The gut and liver are closely interconnected through the portal circulation, forming a complex bidirectional relationship. Disruption of the intestinal barrier facilitates microbial translocation and the influx of bacterial metabolites into the liver, processes that drive both the development and progression of liver diseases [5]. Although the role of gut microbiota in alcohol-related liver disease and infections associated with advanced cirrhosis has long been acknowledged, recent advances in microbiome research and barrier physiology highlight the gut–liver axis as a pivotal component in the pathogenesis of liver disease [6,7].

Gut dysbiosis may be both a consequence and an exacerbating factor in liver disease, with mutual, deleterious interactions observed between the two systems [8]. Via the portal vein, microbial products influence hepatic homeostasis even in physiological conditions, while the liver regulates intestinal microbial composition and barrier function through bile acid secretion, reflecting a dynamic two-way communication [9,10]. Although numerous therapeutic strategies have been explored to correct dysbiosis, most interventions show modest clinical efficacy [11]. An exception is fecal microbiota transplantation (FMT), the infusion of stool from a healthy donor into a recipient’s gastrointestinal tract, which has emerged as a promising intervention to restore microbial diversity and modulate disease progression by rebalancing the gut microbiota [12].

Cirrhosis, the end-stage manifestation of chronic liver injury, remains a major contributor to global morbidity and mortality. It ranks as the 15th leading cause of disability-adjusted life-years globally and the 12th in individuals aged 25 to 49 years, with Europe bearing a disproportionately high burden of premature death [13,14,15]. Each year, chronic liver diseases account for nearly 2 million deaths worldwide, predominantly among men and generate a considerable economic impact, with annual healthcare costs exceeding USD 32.5 billion in the United States alone [13,16].

Histologically, cirrhosis is characterized by widespread nodular regeneration, dense fibrotic septa and parenchymal extinction, resulting in vascular remodeling, portal hypertension and progressive loss of hepatic function [17,18]. Clinically, cirrhosis is considered a terminal disease without liver transplantation, with current preventive strategies limited to surveillance for esophageal varices and hepatocellular carcinoma [19,20].

Given the growing body of evidence highlighting the gut–liver axis as a modifiable factor in chronic liver disease, our study aimed to investigate the clinical and biochemical effects of FMT in patients with compensated liver cirrhosis. Building upon previous findings that suggest potential benefits of microbiota modulation, we conducted a clinical trial to evaluate short-term changes in hepatic function, inflammatory markers and encephalopathy severity following FMT. The following section details the study design, inclusion criteria, procedures and parameters assessed during the one-month follow-up period.

## 2. Materials and Methods

The present research is a prospective study conducted over a three-year period (2023–2025) and includes a total of 19 participants, distributed as follows:

- A total of 6 male patients diagnosed with alcohol-associated cirrhosis who underwent fecal microbiota transplantation;
- A total of 13 male patients with alcohol-associated cirrhosis who served as the control group, receiving standard therapeutic regimens.

Participants were recruited from adult inpatients admitted primarily to the Gastroenterology Department of the County Clinical Emergency Hospital of Sibiu. Patients with liver cirrhosis, diagnosed according to standard international criteria using clinical, laboratory and imaging assessments, were included in the study.

**Figure 1:**
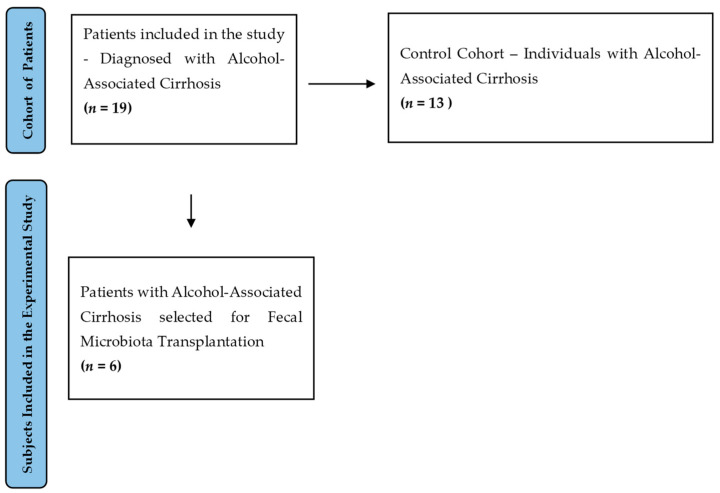
Flow chart illustrating the distribution of subjects included in the study.

Exclusion criteria included individuals younger than 18 years, patients with cirrhosis of non-alcoholic etiology and those with uncertain or unconfirmed diagnoses. The Child–Pugh classification was not an exclusion criterion. Patients with concomitant malignancies, major trauma, hemodynamic or respiratory instability or acute and chronic infections (including HIV, tuberculosis, multidrug-resistant Enterobacteriaceae, cytomegalovirus, parasitic or fungal infections) were also excluded. Furthermore, individuals with severe immunodeficiency, those who declined participation or refused to provide written informed consent, as well as patients in a comatose state or with severely impaired consciousness, were not enrolled. Although gender was not a predefined selection criterion, all enrolled participants were male, which allowed for greater cohort homogeneity by minimizing potential sex-related variability. Following the provision of informed consent, participants were enrolled into specific protocols corresponding to their study group (see Figure 1). A subset of patients presenting features indicative of metabolic dysfunction alongside alcohol-related liver disease (i.e., MetALD) may have been included, mirroring real-world clinical scenarios. Notably, however, none of the enrolled individuals fulfilled the diagnostic criteria for metabolic syndrome, as established by the NCEP ATP III guidelines, requiring the presence of at least three of the following: abdominal obesity, hypertriglyceridemia, low HDL cholesterol, hypertension, or elevated fasting glucose.

Patients selected for FMT (*n* = 6) underwent a comprehensive clinical and paraclinical evaluation, including physical examination, collection of biological samples (blood and stool) and imaging assessments such as ultrasound and elastography. A complete colonoscopy was performed up to the cecum, followed by the administration of fecal material from a healthy donor. Following transplantation, patients were scheduled for follow-up evaluations at approximately 7 days (±2 days) and at 1 month post-procedure. The exact timing of assessments varied based on patient availability and logistical constraints. Each follow-up included a clinical examination, ultrasound imaging, and blood sampling. Elastography was repeated during the second evaluation. Inclusion in this group required that patients had not used antibiotics in the three months prior to the intervention and had no recent history of hospitalization.

Throughout the study, both alcohol consumption and the use of any antibiotics, including rifaximin, were strictly prohibited. All patients had a documented history of chronic alcohol consumption consistent with alcohol-related liver disease at diagnosis. However, sustained abstinence from alcohol was a strict inclusion criterion at enrollment, and none of the participants were actively drinking at study initiation. Eligibility was limited to patients who demonstrated strong compliance and reliability, evaluated through prior clinical follow-up, caregiver support and willingness to adhere to study requirements. Alcohol abstinence was confirmed at each stage by patient self-report and caregiver or family confirmation. No cases of relapse occurred during screening or follow-up, reflecting the careful selection of individuals with a proven history of abstinence and adherence to medical recommendations. This strategy minimized the risk of confounding due to resumed alcohol use, which could otherwise have significantly influenced liver-related outcomes.

The control group (*n* = 13) received standard-of-care therapy (including a normocaloric, normoproteic, normolipidic and normoglucidic diet, beta-blockers, hepatoprotective adjuvants, diuretics) and underwent baseline evaluations (designated as time zero), including clinical examination, imaging, elastography and laboratory tests, followed by repeat assessments after approximately one month. As with the intervention group, some variability in follow-up timing was allowed. Alcohol consumption and antibiotic use were restricted throughout the study, with the exception of rifaximin, which was permitted in the control group.

Fecal donors were selected from among both patient relatives and unrelated healthy volunteers. Preference was given to young individuals without comorbidities, who provided written informed consent. All donors underwent rigorous clinical and paraclinical screening to minimize the risk of contamination, as recommended by other studies in the literature on this subject [21,22]. Blood samples were collected for general assessment, including complete blood count, general biochemical and coagulation tests and also specific investigations to exclude HIV, hepatitis and autoimmune diseases. Additionally, stool samples were examined for occult bleeding, as well as for bacterial, parasitic and other infectious agents. Screening also included a mandatory questionnaire to identify potential epidemiological risks capable of transmitting infectious or other diseases to immunocompromised recipients with cirrhosis.

Numerous methods for preparing fecal material for transplantation have been described in the literature, all aiming to optimize microbial viability, minimize the risk of contamination, and ensure patient safety [23,24]. On the procedure day, donors prepared the fecal material following a standardized protocol provided at the time of donation approval. This preparation model was adapted from the protocol routinely employed at the Sibiu Gastroenterology Clinic for patients with *Clostridioides difficile* infection and was meticulously followed throughout. Specifically, fecal material was collected on the morning of the scheduled transplantation and was used within a maximum of six hours, as extended storage would compromise its effectiveness. A minimum of 70 g of stool was obtained and subsequently mixed with 250 mL of 0.9% sodium chloride solution in a sterile container, with continuous homogenization for at least 3 minutes. The resulting mixture was then filtered through 2–3 layers of sterile gauze and the filtrate was transported to the hospital for transplantation. Microbial composition analysis of fecal samples and FMT preparations was not performed in this pilot study, as the design primarily focused on clinical and biochemical endpoints.

The colonoscopic procedure was performed using standard protocols, including bowel preparation with polyethylene glycol solutions. Analgesia and sedation were administered uniformly to ensure patient comfort. To optimize transplant retention and therapeutic efficacy, patients were instructed to refrain from defecating for a minimum of two hours post-procedure. No concomitant medications were permitted that could potentially confound the study results.

Hepatic encephalopathy was assessed using the EncephalApp—Stroop Test, a method widely recognized for its high sensitivity in detecting minimal hepatic encephalopathy, which is often challenging to identify clinically. Additionally, staging of encephalopathy was performed by correlating the cognitive test results with clinical findings, in accordance with the West Haven classification [25,26,27].

For statistical analysis, continuous variables were reported as medians and interquartile ranges (IQRs). Statistical tests were selected based on the distribution and nature of the data. A 95% confidence interval was applied throughout and *p*-values < 0.05 were considered statistically significant. Data analysis was conducted using SPSS software, version 24.

## 3. Ethical Implications

From both ethical and legal perspectives, the conduct of clinical trials requires strict adherence to regulatory standards, including prior approval by institutional ethics committees and registration in an internationally recognized clinical trial database. In alignment with these principles, the present study received favorable ethical clearance from the Ethics Committee of the County Clinical Emergency Hospital of Sibiu (Approval No. 1968, issued on 30 January 2023). Additionally, ethical approval was granted by the Ethics Committee of “Lucian Blaga” University of Sibiu (Approval No. 18, dated 10 February 2023). The trial was subsequently registered in the United States-based international clinical trial registry (ClinicalTrials.gov), with full protocol details and outcomes publicly available under the identifier NCT06478602 (Institutional Code: 3505/15.02.2023), accessible at https://clinicaltrials.gov/study/NCT06478602 (accessed on 20 June 2025).

All participants enrolled in the study were required to sign a standardized informed consent form, which had been carefully developed to ensure full transparency regarding the nature of the clinical trial, its objectives, potential benefits and associated risks. These consent forms were included in the documentation submitted for ethical approval, along with the donor screening questionnaires. Given the sensitive nature of the medical and personal data collected, donor participants were additionally required to complete a General Data Protection Regulation (GDPR) compliance section. This provision was implemented to safeguard donor privacy and to provide explicit information regarding data usage, storage and confidentiality throughout the study. The research was conducted over the full three-year period without any adverse events or circumstances necessitating suspension or early termination of the trial.

## 4. Results

### 4.1. Basic Characteristics of the Study Group (FMT) Compared to the Control Group

Within this study, multiple clinical and biological variables were compared between the study group (*n* = 6) and the control group (*n* = 13). Statistically significant differences were analyzed using the Mann–Whitney U test for continuous variables and Fisher’s exact test for categorical variables.

The median age of participants was 54 years (IQR: 52–57) in the study group, compared to 51 years (IQR: 44.5–62) in the control group, with no statistically significant difference observed (*p* = 0.539). Likewise, no significant differences were observed in body mass index (BMI), where the *p*-value was 0.273.

Residential distribution did not differ significantly between groups. In the intervention group, 83.3% of patients resided in urban areas, compared to 38.5% in the control group; however, this difference did not reach statistical significance.

Liver elasticity (E/kPa) was 25.95 kPa (IQR: 23.8–27) in the study group and 22.55 kPa (IQR: 21.65–23.9) in the control group, with no statistically significant difference observed (*p* = 0.161). Similarly, the controlled attenuation parameter (CAP, measured in decibels per meter) recorded values of 180.5 dB/m (IQR: 166–210) in the study group and 175.5 dB/m (IQR: 144.5–200.5) in the control group, without significant statistical difference (*p* = 0.831) (Table 1).

### 4.2. Evolution of Biological and Fibrosis Markers One Week After Fecal Microbiota Transplantation in the Study Group

(a). Hepatic fibrosis markers and major hepatic syndrome indicators

One week after FMT, a general trend toward improvement in hepatic parameters was observed. Although most changes did not reach statistical significance, two key variables showed significant improvement: liver stiffness, as measured by elastography, demonstrated a statistically significant reduction and the controlled attenuation parameter (CAP, expressed in dB/m) also decreased significantly following FMT (Table 2).

In contrast, variations in standard biochemical markers, including total bilirubin, cholinesterase, gamma-glutamyltransferase (GGT), total protein levels, transaminases, prothrombin time and international normalized ratio (INR), were modest and did not achieve statistical significance (Table 2).

(b). Extended biochemical markers

Upon evaluation of biochemical marker variations one week after FMT, statistically significant changes were identified in a limited number of parameters. Serum albumin levels exhibited a significant decrease compared to baseline values (*p* = 0.043), while gamma-globulin levels also declined significantly (*p* = 0.046). In contrast, no statistically significant changes were observed in other markers, including uric acid, amylase, cholesterol, creatinine and alpha-1 globulins (*p* > 0.05), indicating that these variables remained largely unaffected by FMT at the one-week follow-up (Table 3). Although leukocyte counts remained unchanged one week post-FMT, a more substantial reduction was observed in C-reactive protein levels (13.2 [6.6–27.3] vs. 4.5 [2.8–7.4], *p* = 0.116), potentially reflecting a decline in systemic inflammation linked to cirrhosis and suggesting a possible early amelioration of bacterial translocation.

(c). One-week clinical evolution

Comparative assessment of clinical parameters before and one week after FMT suggested potentially favorable outcomes in patients with liver cirrhosis, with a particularly significant improvement in the severity of hepatic encephalopathy. Prior to FMT, 83.3% of patients presented with grade 1 encephalopathy and 16.6% with grade 2. At one week post-intervention, 83.3% of patients no longer exhibited clinical signs of encephalopathy (grade 0), while the remaining 16.6% presented with only grade 1. This improvement was statistically significant (*p* = 0.014), suggesting an early therapeutic effect of FMT on hepatic encephalopathy manifestations. In contrast, no changes were observed in the distribution of Child–Pugh classes following the intervention, with differences remaining statistically non-significant (Table 4).

### 4.3. Evaluation of the Changes in Biological and Liver Fibrosis Markers One Month After FMT Administration in the Study Group

(a). Hepatic fibrosis markers and major liver syndrome indicators

**Figure 2:**
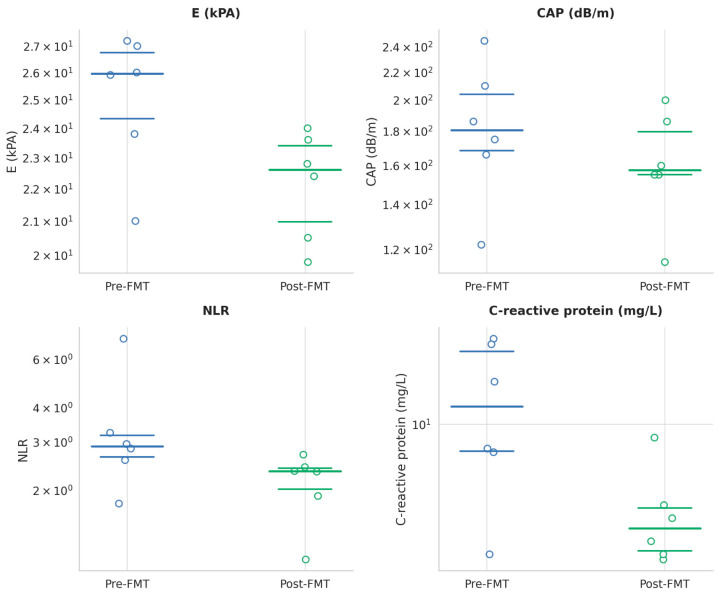
Paired values of C-reactive protein (mg/L), Neutrophil-Lymphocyte Ratio, liver stiffness (E, kPa), and controlled attenuation parameter (CAP, dB/m) before and after fecal microbiota transplantation (FMT)-1 month. All variables are statistically significant.

The analysis of hepatic parameters one month following FMT revealed both statistically significant and non-significant changes. Liver stiffness, assessed via elastography, showed a statistically significant reduction from a pre-intervention median of 25.95 kPa (IQR: 23.8–27.0) to 22.6 kPa (IQR: 19.9–23.4) at one month post-FMT, maintaining the improvement observed at the one-week follow-up. Similarly, CAP demonstrated a significant decrease, from a median of 180.5 dB/m (IQR: 166–210) prior to transplantation to 157.5 dB/m (IQR: 125–179.5) after one month (*p* = 0.025) (Table 5 and Figure 2).

Conversely, no significant differences were observed in the FIB-4 and APRI scores between baseline and one week after the intervention, both yielding *p*-values of 0.917. In addition, no statistically significant changes (*p* > 0.05) were found in key biochemical markers, including albumin, total bilirubin, cholinesterase, gamma-glutamyltransferase (GGT), total proteins, AST, ALT, prothrombin time and INR. Notably, the significant reductions in liver stiffness and CAP values observed at one week were sustained at the one-month evaluation.

(b). Extended biological markers

At one month post-FMT, the analysis of extended biological markers revealed statistically significant changes in several parameters. C-reactive protein (CRP) levels decreased significantly from a median of 13.2 mg/L (IQR: 6.65–27.3) at baseline to 2.30 mg/L (IQR: 1.40–5.46) post-FMT (*p* = 0.043), indicating a marked reduction in systemic inflammatory activity. Likewise, the neutrophil-to-lymphocyte ratio (NLR) significantly declined from 2.9 (IQR: 2.7–3.1) to 2.35 (IQR: 1.51–2.56) (*p* = 0.043), suggesting a potential normalization of the immune-inflammatory balance following the intervention. In contrast, carcinoembryonic antigen (CEA) levels increased significantly, from a median of 2.3 ng/mL (IQR: 1.9–2.8) before transplantation to 2.82 ng/mL (IQR: 1.65–5.3) at one month post-procedure, as presented in Table 6 and Figure 2.

BMI also exhibited a statistically significant increase, rising from a median of 24.4 kg/m^2^ (IQR: 21.5–26.9) to 25.46 kg/m^2^ (IQR: 20.5–28.24) one month after FMT (*p* = 0.046), which may reflect an early improvement in nutritional status. Other biological markers, including amylase, total cholesterol, HDL cholesterol, LDL cholesterol and immunoglobulin levels (IgA, IgG, IgM), did not demonstrate statistically significant changes (*p* > 0.05), as detailed in Table 6.

(c). Clinical Evolution at 1 Month

The severity of hepatic encephalopathy demonstrated statistically significant improvements one month following FMT, indicating substantial neuropsychiatric recovery. The proportion of patients without clinical signs of encephalopathy (grade 0) increased from 0% at baseline to 83.3% after the intervention (*p* = 0.014), underscoring the therapeutic potential of FMT. Concurrently, the proportion of patients with grade 1 encephalopathy decreased from 83.3% to 16.6%, while those with grade 2 encephalopathy were no longer present (a reduction from 16.6% to 0%). These improvements were statistically significant (*p* < 0.05), as detailed in Table 7.

Notably, when compared to the one-week post-transplant assessment, the encephalopathy status remained stable, suggesting that the neuropsychiatric benefits achieved early after FMT were maintained over time. This sustained response supports the hypothesis of a durable and stable therapeutic effect induced by the intervention. An additional clinically relevant observation was the reclassification of one patient from Child–Pugh Class B to Class A at one month post-FMT, reflecting a notable improvement in overall hepatic function. This transition may suggest either partial functional recovery of hepatic parenchyma or an enhanced compensatory response of liver function following FMT administration.

### 4.4. Baseline Characteristics of the Study Group Compared to the Control Group One Month After FMT

**Figure 3:**
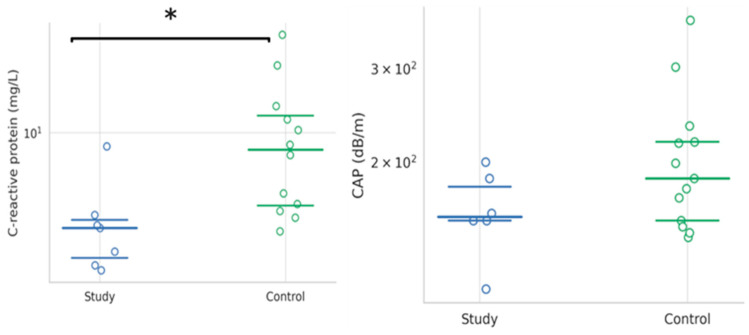
Values of C-reactive protein (mg/L) and controlled attenuation parameter (CAP, dB/m) 1 month after fecal microbiota transplantation (FMT) between study group and control group. *p*-values < 0.05 are flagged with *.

The comparative evaluation of clinical and biological parameters between the intervention group and the control group revealed statistically significant differences in only two variables: serum sodium concentration and CRP levels. Specifically, serum sodium levels were significantly lower in the FMT group, with a median value of 136 mmol/L, compared to 138.5 mmol/L in the control group (*p* = 0.029). Likewise, CRP levels were notably reduced in the intervention group, with a mean value of 2.3 mg/L vs. 7.7 mg/L in the control group (*p* = 0.034), suggesting a diminished systemic inflammatory response following fecal microbiota transplantation (Figure 3). No statistically significant differences were observed between the two groups with respect to hepatobiliary markers, including FIB-4 and APRI scores, liver stiffness measured by elastography and CAP (Table 8 and Figure 3).

Liver stiffness and CAP showed statistically significant improvements at both one week and one month following FMT, relative to baseline pre-intervention values. These favorable changes were sustained throughout the one-month follow-up period, indicating a potentially durable hepatic response to FMT. Nonetheless, although a positive trend was observed in the intervention group when compared to the control group receiving standard treatment, the intergroup differences at one month post-intervention did not reach statistical significance (Table 8).

## 5. Discussion

The initial step in interpreting the study outcomes involved a detailed characterization of the dataset, particularly the comparison between the intervention and control groups (Table 1). This evaluation is essential to detect and mitigate any potential imbalances or biases in patient selection, which is especially relevant in alcohol-related liver cirrhosis, where inclusion timing may be influenced by episodes of acute decompensation. Such episodes can significantly alter both clinical presentation and biochemical parameters, potentially confounding the interpretation of intervention effects. Therefore, rigorous baseline assessment is a prerequisite for ensuring the internal validity of the study.

Biological markers, particularly those associated with inflammatory status or hepatocellular injury, are highly sensitive to external influences and prone to fluctuation. Encouragingly, baseline comparisons revealed no statistically significant differences between the study groups in these parameters, including demographic factors such as age, place of residence and BMI. This homogeneity strengthens the reliability of subsequent intergroup comparisons.

One week following FMT, notable improvements were observed in liver stiffness and steatosis, as reflected by reductions in elastography and CAP values, respectively. Although these changes did not result in categorical shifts in fibrosis or steatosis grades, the quantitative reductions were statistically significant, with liver stiffness approaching the F3 fibrosis threshold. The magnitude of steatosis reduction warrants further consideration, particularly in relation to circulating lipid markers: total cholesterol, HDL and LDL, all of which exhibited non-significant but downward trends. However, the concurrent decline in HDL levels does not imply a clear clinical benefit, underscoring the complexity of interpreting metabolic shifts post-FMT. A plausible mechanism underlying these hepatic improvements may involve attenuation of systemic inflammation. Although this hypothesis is supported by the prior literature, definitive evidence remains limited, in part due to the variability and limited precision of conventional inflammatory biomarkers [28,29,30]. Moreover, elastography results should be interpreted with caution, especially given that variations may occur in some cases [31].

Additional inflammatory markers, including CRP, ferritin, immunoglobulins (IgA, IgM, IgG), amylase and CA 19-9, also trended downward, although these changes did not achieve statistical significance. Notably, CA 19-9, which can be elevated in benign inflammatory conditions, showed a reduction, potentially reflecting improved inflammatory control [32,33].

Liver transaminases (AST and ALT) remained stable, with no significant changes observed. This was expected given the compensated nature of the cirrhosis in the study population and the absence of acute hepatocellular injury at baseline. Consequently, the utility of transaminases as markers of FMT efficacy in this context is limited. Similarly, FIB-4 and APRI scores, primarily used as negative predictive tools, did not significantly change, which aligns with their limited sensitivity to short-term functional shifts.

Decreasing trends in total protein, vitamin B12 and folic acid levels may reflect altered intestinal absorption dynamics following microbiota modulation. Nevertheless, this area remains largely unclear, primarily due to the contradictory data reported in the literature. Some studies have suggested a strong correlation between serum levels of vitamin B12 and the diversity of the gut microbiota, and the restoration of microbial balance should, in theory, lead to an increase in circulating concentrations of vitamins B12 and B9. Research conducted on animal models has also demonstrated elevated levels of vitamin B12. In this context, the only plausible hypothesis that might account for the discrepancy with existing data relates to absorption mechanisms; however, the underlying pathophysiological processes require further investigation [34,35]. A particularly intriguing finding was reported in the study by Zabolotneva et al., which demonstrated an association between obesity induced in mice and the depletion of vitamin B12 levels. In line with these experimental data, our study revealed a downward trend in serum vitamin B12 concentration (at one month post-FMT), which was correlated with a marked weight gain among patients in the experimental group. This observation suggests a potential causal relationship and may provide an additional explanation for the pronounced decline in vitamin B12 levels [36]

The hematological profile following FMT is of particular interest given the scarcity of detailed reports, as thrombocytopenia showed a non-significant trend toward improvement, whereas the literature has documented a case of reproducible, dramatic post-FMT thrombocytopenia, suggesting a potential but poorly understood immune-related hematologic mechanism [37].

Among the most clinically relevant findings was the marked improvement in hepatic encephalopathy at one week post-FMT. This effect is consistent with current studies reported in the literature [38,39,40]. Importantly, no cases of encephalopathy worsening have been reported following FMT. The mechanism remains uncertain, but is likely multifactorial, possibly involving improved hepatic clearance, reduced ammonia production and altered gut toxin absorption [41,42,43].

Recent trials have highlighted that FMT can beneficially reshape the gut microbiota in cirrhosis and hepatic encephalopathy, with consistent enrichment of short-chain fatty acid–producing taxa such as Lachnospiraceae and Ruminococcaceae [23]. These bacterial families are associated with improved intestinal barrier integrity and reduced systemic inflammation, thereby modulating the gut–liver axis and decreasing the risk of HE recurrence [39]. Moreover, higher pre-FMT abundance of Lachnospiraceae has been identified as a predictor of successful donor engraftment and reduced recurrence rates, underscoring the key role of these beneficial taxa in mediating clinical outcomes after FMT [40].

Of particular interest is the sustained improvement in encephalopathy observed at one month post-FMT, even in the absence of rifaximin. This suggests that FMT may induce long-term modulation of gut microbiota composition, leading to persistent neuropsychiatric benefits. The absorption changes observed for vitamins B12 and B9 may extend to gut-derived neurotoxins, offering a potential mechanistic explanation for the reduced encephalopathy burden.

Comparative analysis with the control group further supports the positive impact of FMT. While the small sample size limits statistical power, significant intergroup differences were observed in CRP levels, suggesting a lower inflammatory burden in the FMT group. Although serum sodium levels were also significantly lower in the intervention group, the clinical implications of this finding remain limited.

Encephalopathy was notably less severe in the FMT group compared to controls, reinforcing the therapeutic effect observed within the intervention group itself. However, other markers such as CAP and liver elasticity, although improved from baseline, did not differ significantly between groups at one month post-intervention, likely due to sample size constraints.

## 6. Limitations

The main limitation of this trial is the absence of microbiota composition analysis, as neither the donor material nor the recipients’ fecal samples were characterized before and after FMT. Consequently, it was not possible to correlate the observed clinical improvements with specific microbial shifts or to assess the potential role of particular bacterial taxa in modulating the gut–liver axis. This omission reduces the mechanistic depth of our findings and should be addressed in future studies through integrated microbial and metabolomic profiling.

Another important limitation is the small sample size and the single-center design, which restrict the generalizability of our results. The limited number of participants may also have prevented us from detecting certain clinically relevant differences between groups. In addition, the follow-up period was restricted to one month, precluding conclusions on the long-term efficacy and safety of FMT in alcohol-associated cirrhosis. Larger, multicenter trials with extended follow-up are needed to validate and expand upon our findings.

## 7. Conclusions

This prospective study provides emerging evidence that fecal microbiota transplantation (FMT) may serve as a safe and effective adjunctive therapy in patients with alcohol-associated cirrhosis. Clinically significant improvements were observed in the severity of hepatic encephalopathy, as well as in liver stiffness, steatosis and systemic inflammation as early as one week post-FMT, with these effects sustained at one month. The marked reduction in neuropsychiatric symptoms and inflammatory markers such as CRP and NLR highlights the potential of microbiota modulation to influence both hepatic and systemic outcomes.

The absence of significant adverse events and the stability of clinical parameters further reinforce FMT’s favorable safety profile in this population.

Given the limited cohort size and single-center design, the present findings should be interpreted with caution. However, they offer a compelling rationale for future large-scale, multicenter studies to confirm the therapeutic utility of FMT in liver cirrhosis and to elucidate its mechanistic underpinnings.

## Figures and Tables

**Table 1:** Basic characteristics of the study group (FMT) compared to the control group.

Variable	Study Group (*n* = 6)	Control Group (*n* = 13)	*p*-Value
Age (years)	54 (52–57)	51 (44.5–62)	0.539
Urban Residence (%)	5 (83.3%)	5 (38.5%)	0.141
Body Mass Index (BMI, kg/m^2^)	24.4 (21.5–26.9)	25.55 (23.6–27.45)	0.273
Serum Amylase (U/L)	81.5 (64–93)	65.5 (48.5–98.5)	0.482
Total Bilirubin (mg/dL)	4.18 (1.33–7.65)	1.785 (0.515–3.095)	0.096
Creatinine (mg/dL)	0.63 (0.58–0.69)	0.875 (0.59–1.07)	0.219
Blood Glucose (mg/dL)	102.5 (89–117)	98.5 (94.5–113)	0.763
Sodium (mmol/L)	136.5 (136–137)	138 (136–139.5)	0.569
Potassium (mmol/L)	4.46 (3.91–4.61)	4.05 (3.91–4.215)	0.512
Chloride (mmol/L)	103.6 (99–106)	102.2 (98.5–104.6)	0.925
C-Reactive Protein (CRP, mg/L)	13.19 (6.46–35.1)	6.25 (2.88–19.5)	0.430
Aspartate Aminotransferase (TGO, U/L)	39 (33–52)	47.5 (25–111.5)	0.483
Alanine Aminotransferase (TGP, U/L)	26 (24–30)	27 (19–40)	0.693
Urea (mg/dL)	27 (22–32)	33.5 (20.5–43.5)	0.399
Prothrombin Time (PT, seconds)	16 (13.3–18.3)	13.5 (12.4–15.3)	0.190
International Normalized Ratio (INR)	1.4 (1.19–1.57)	1.175 (1.07–1.36)	0.206
Activated Partial Thromboplastin Time (APTT, seconds)	38.45 (30.6–43.1)	35.3 (31.35–39.4)	0.512
APTT Ratio	1.285 (1.02–1.44)	1.175 (1.045–1.31)	0.542
Leukocytes (cells/μL)	7.14 (6.86–8.1)	7.71 (5.925–10.315)	0.861
Erythrocytes (cells/μL)	4.265 (3.68–4.49)	4.44 (3.945–4.83)	0.861
Hemoglobin (g/dL)	13.45 (12.6–14.9)	13.6 (11.95–14.85)	0.430
Hematocrit (%)	38.95 (35.8–42.4)	38.3 (35.4–42.55)	0.334
Mean Corpuscular Volume (MCV, fL)	93.25 (91.3–94.4)	89.45 (86.45–92.5)	0.160
Mean Corpuscular Hemoglobin (MCH, g/dL)	32.9 (32.2–33.2)	31.05 (30.6–32.4)	0.661
Mean Corpuscular Hemoglobin Concentration (MCHC, g/dL)	35.15 (34.1–35.6)	34.65 (34.05–35.45)	0.826
Platelets (cells/μL)	145 (108–195)	131 (86–157)	0.335
Neutrophils (cells/μL)	4.745 (3.66–5.37)	5.31 (3–6.5)	1.000
Lymphocytes (cells/μL)	1.64 (1.42–1.66)	1.78 (1.37–2.315)	0.599
Monocytes (cells/μL)	0.77 (0.55–0.96)	0.715 (0.55–0.88)	0.861
Basophils (cells/μL)	0.04 (0.04–0.08)	0.06 (0.05–0.075)	0.594
Eosinophils (cells/μL)	0.2 (0.18–0.21)	0.115 (0.065–0.23)	0.379
Neutrophil-to-Lymphocyte Ratio (NLR)	2.8 (2.5–3.2)	2.8 (1.4–4.3)	0.430
Encephalopathy (%)			
Grade 0	0 (0%)	4 (30.8%)	0.299
Grade 1	5 (83.3%)	7 (53.8%)	
Grade 2	1 (16.6%)	2 (15.4%)	
Child–Pugh Class			
Class A	2 (33,3%)	3 (23.1%)	0.698
Class B	3 (50%)	9 (69.2%)	
Class C	1 (16,7%)	1 (7.7%)	
FIB-4 Index	3.355 (2.58–4)	3.755 (2.18–13.73)	0.160
APRI Score	0.855 (0.677–1.131)	1.047 (0.554–5.494)	0.161
Hepatic Elasticity (E, kPa)	25.95 (23.8–27)	22.55 (21.65–23.9)	0.161
Controlled Attenuation Parameter (CAP, dB/m)	180.5 (166–210)	175.5 (144.5–200.5)	0.831

**Table 2:** Dynamics of fibrosis and biochemical liver markers one week after fecal microbiota transplantation.

Variable	Pre-FMT	1 Week After FMT	*p*-Value
FIB-4	3.355 (2.58–4)	2.46 (3.22–3.67)	0.600
APRI	0.85 (0.67–1.13)	0.85 (0.67–0.92)	0.917
Liver Elasticity (E, kPa)	25.95 (23.8–27)	21.85 (20.4–22.9)	**0.028**
Controlled Attenuation Parameter (CAP, dB/m)	180.5 (166–210)	169.5 (158–199)	**0.027**
Albumin (g/L)	38.30 (33.50–39.55)	35.85 (29–38.1)	**0.043**
Total Bilirubin	4.18 (1.33–7.65)	3.38 (1.67–4.61)	0.414
Cholinesterase	5870.5 (4210.0–6718.0)	5675.5 (3805.5–6946.0)	0.414
Gamma-Glutamyl Transferase (GGT)	117.5 (83.5–211.0)	81 (73–130.5)	0.102
Total Proteins	7.35 (7.00–7.70)	7.2 (7–7.30)	0.655
Aspartate Aminotransferase (TGO)	39 (33–52)	41 (35.0–44.5)	0.986
Alanine Aminotransferase (TGP)	26 (24–30)	25 (22.5–31.0)	0.963
Prothrombin Time	16 (13.3–18.3)	14.85 (13.70–16.90)	0.688
International Normalized Ratio (INR)	1.4 (1.19–1.57)	1.19 (1.14–1.31)	0.414

**Table 3:** Dynamics of other biological markers one week after FMT.

Variable	Pre-FMT	1 Week After FMT	*p*-Value
Uric Acid—mg/dL	4.7 (3.9–5.0)	4.2 (3.9–4.8)	0.917
Amylase—U/L	81.5 (70–89)	69 (50–88)	0.528
Cholesterol—mg/dL	142.5 (119.5–160)	140 (123–161)	0.344
Creatinine—mg/dL	0.6 (0.6–0.7)	0.63 (0.615–0.655)	0.598
Alpha-1 Globulins—g/dL	0.3 (0.2–0.3)	0.24 (0.21–0.24)	0.102
Alpha-2 Globulins—g/dL	0.7 (0.6–0.7)	0.62 (0.52–0.73)	0.115
Beta-1 Globulins—g/dL	0.6 (0.6–0.6)	0.56 (0.51–0.60)	0.527
Beta-2 Globulins—g/dL	0.5 (0.5–0.5)	0.49 (0.43–0.59)	0.465
Gamma Globulins—g/dL	2.0 (1.7–2.4)	1.78 (1.55–1.925)	**0.046**
Albumin/Globulin Ratio	0.9 (0.7–1.1)	0.92 (0.795–1.08)	0.080
Serum Iron (Sideremia)—µg/dL	128.5 (119–150.5)	111 (89–139)	0.644
Alkaline Phosphatase—U/L	116.5 (86.5–158)	124 (85.5–161)	0.686
HDL Cholesterol—mg/dL	39.5 (15–56.5)	35 (24.5–46.5)	0.647
LDL Cholesterol—mg/dL	88.6 (74.9–112.4)	86.1 (80.4–98.7)	0.225
IgA—mg/dL	795 (616–921.5)	673.5 (594.5–797)	0.833
IgG—mg/dL	2041.5 (1779–2159)	1990 (1670.5–2185)	0.463
IgM—mg/dL	154 (132.5–174.5)	146 (127.5–162)	0.115
Sodium (Na)—mEq/L	136.5 (136–137)	135 (134–137)	0.463
Potassium (K)—mEq/L	4.5 (4.2–4.6)	4.4 (4.24–4.50)	0.753
C-Reactive Protein (CRP)—mg/L	13.2 (6.6–27.3)	4.5 (2.8–7.4)	0.116
Triglycerides—mg/dL	73.5 (68–90.5)	75 (59–78)	0.293
Leukocytes—×10^3^/µL	7.1 (6.9–7.7)	7.46 (6.8–8.2)	0.833
Hemoglobin—g/dL	13.5 (12.7–14.6)	13.55 (12.20–14.70)	0.344
Platelets—×10^3^/µL	133 (120.5–176)	154 (147–157.5)	0.916
Neutrophil-to-Lymphocyte Ratio (NLR)	2.9 (2.7–3.1)	2.28 (1.765–2.62)	0.753
Erythrocyte Sedimentation Rate (ESR/VSH)—mm/h	33 (26–39.5)	22.5 (15–37.5)	0.075
Folic Acid—ng/mL	7.5 (5.9–10)	5.70 (4.38–7.80)	0.249
Alpha-Fetoprotein (AFP)—ng/mL	4.1 (3.5–8.9)	7.15 (4.10–8.15)	0.581
CA 19-9—U/mL	31.6 (18.3–41.7)	14.89 (8.71–29.98)	0.345
Carcinoembryonic Antigen (CEA)—ng/mL	2.3 (1.9–2.8)	2.5 (1.45–2.65)	0.528
Ferritin—ng/mL	270.7 (157.4–346.9)	173.85 (51.1–269.9)	0.463
Vitamin B12—pg/mL	870 (314–1330)	753.7 (328.4–997)	0.173
Body Mass Index (BMI)—kg/m^2^	24.4 (21.5–26.9)	24.6 (20.6–27)	0.106

**Table 4:** Dynamics of clinical indicators in the progression of liver cirrhosis one week after fecal microbiota transplantation.

Variable	Pre-FMT	1 Week After FMT	*p*-Value
Child–Pugh Class			
Class A	2 (33.3%)	2 (33.3%)	1.000
Class B	3 (50%)	3 (50%)	
Class C	1 (16.7%)	1 (16.7%)	
Grade of Encephalopathy			
Grade 0	0 (0%)	5 (83.3%)	**0.014**
Grade 1	5 (83.3%)	1 (16.6%)	
Grade 2	1 (16.6%)	0 (0%)	

**Table 5:** Dynamics of fibrosis markers and liver biochemical parameters one month after fecal microbiota transplantation.

Variable	Pre-FMT	1 Month Post-FMT	*p*-Value
FIB-4	3.35 (2.58–4)	3.25 (2.2–3.7)	0.917
APRI	0.85 (0.67–1.13)	0.87 (0.56–1.13)	0.917
E (kPa)	25.95 (23.8–27)	22.6 (19.9–23.4)	***0.027***
CAP (dB/m)	180.5 (166–210)	157.5 (125–179.5)	***0.025***
Albumin	38.3 (33.5–39.55)	38 (28.25–39.95)	0.500
Total bilirubin	4.18 (1.33–7.65)	2.14 (1.07–4.23)	0.345
Cholinesterase	5870.5 (4210–6718)	5794 (3895–8292)	0.893
GGT	117.5 (83.5–211)	95.5 (42.75–137)	0.917
Total proteins	7.35 (7.00–7.70)	7.55 (6.575–8.025)	0.833
TGO	39 (33–52)	39 (28.75–47.25)	0.463
TGP	26 (24–30)	29.5 (17.0–30.75)	0.753
Prothrombin time	16 (13.3–18.3)	14.1 (12.85–17.4)	0.753
INR	1.4 (1.19–1.57)	1.24 (1.13–1.56)	0.753

**Table 6:** Dynamics of extended biological markers at one month post-FMT.

Variable	Pre-FMT	1 Month Post-FMT	*p*-Value
Uric acid—mg/dL	4.7 (3.9–5)	4.45 (4.3–5.12)	0.463
Amylase—U/L	81.5 (70–89)	87 (70.25–88.75)	0.753
Cholesterol—mg/dL	142.5 (119.5–160)	150.0 (110.5–201.5)	0.249
Creatinine—mg/dL	0.6 (0.6–0.7)	0.68 (0.6–0.72)	***0.027***
Alpha 1-globulins—g/dL	0.3 (0.2–0.3)	0.23 (0.21–0.23)	0.223
Alpha 2-globulins—g/dL	0.7 (0.6–0.7)	0.69 (0.68–0.72)	0.715
Beta 1-globulins—g/dL	0.6 (0.6–0.6)	0.62 (0.58–0.63)	1.000
Beta 2-globulins—g/dL	0.5 (0.5–0.5)	0.48 (0.48–0.59)	0.221
Gamma-globulins—g/dL	2 (1.7–2.4)	1.75 (1.66–2.21)	0.893
Albumin/globulin ratio	0.9 (0.7–1.1)	1.07 (0.89–1.11)	0.078
Serum iron—µg/dL	128.5 (119–150.5)	133 (104–141)	0.345
Alkaline phosphatase—U/L	116.5 (86.5–158)	108.5 (71.5–143)	0.345
HDL cholesterol—mg/dL	39.5 (15–56.5)	39 (36–52.5)	0.173
LDL cholesterol—mg/dL	88.6 (74.9–112.4)	93.5 (53.7–118.3)	0.500
IgA—mg/dL	795 (616–921.5)	733 (604–1006)	0.080
IgG—mg/dL	2041.5 (1779–2159)	1797 (1668–2237)	0.684
IgM—mg/dL	154 (132.5–174.5)	155 (134–158)	0.500
Sodium (Na)—mEq/L	136.5 (136–137)	136 (135–136.5)	0.524
Potassium (K)—mEq/L	4.5 (4.2–4.6)	4.32 (4.07–4.60)	0.917
C-reactive protein (CRP)—mg/L	13.2 (6.65–27.3)	2.30 (1.40–5.46)	***0.043***
Triglycerides—mg/dL	73.5 (68–90.5)	68.5 (45.0–79.5)	0.345
Leukocytes—×10^3^/µL	7.1 (6.9–7.7)	7.38 (6.53–7.73)	0.753
Hemoglobin—g/dL	13.5 (12.7–14.6)	14.2 (13.1–14.9)	0.461
Platelets—×10^3^/µL	133 (120.5–176)	143.5 (107.5–168)	0.600
Neutrophil-to-lymphocyte ratio (NLR)	2.9 (2.7–3.1)	2.35 (1.51–2.56)	***0.043***
Erythrocyte sedimentation rate (ESR)—mm/h	33 (26–39.5)	18 (13–42.5)	0.279
Folic acid—ng/mL	7.5 (5.9–10)	6.14 (2.7–9)	0.225
Alpha-fetoprotein (AFP)—ng/mL	4.1 (3.5–8.9)	4.70 (3.45–8.7)	0.102
CA 19-9—U/mL	31.6 (18.3–41.7)	19.59 (7.19–86.6)	0.893
Carcinoembryonic antigen (CEA)—ng/mL	2.3 (1.9–2.8)	2.82 (1.65–5.3)	***0.043***
Ferritin—ng/mL	270.7 (157.4–346.9)	94.20 (48.4–309)	0.345
Vitamin B12—pg/mL	870 (314–1330)	490 (304.9–867.5)	0.225
Body mass index (BMI)—kg/m^2^	24.4 (21.5–26.9)	25.46 (20.5–28.24)	***0.046***

**Table 7:** Dynamics of clinical indicators reflecting the progression of liver cirrhosis one month after FMT.

Variable	Pre-FMT	1 Month Post-FMT	*p*-Value
*Child–Pugh Class*			
Class A	2 (33.3%)	3 (50%)	0.314
Class B	3 (50%)	2 (33.3%)	
Class C	1 (16.7%)	1 (16.7%)	
*Grade of Encephalopathy*			
Grade 0	0 (0%)	5 (83.3%)	***0.014***
Grade 1	5 (83.3%)	1 (16.6%)	
Grade 2	1 (16.6%)	0 (0%)	

**Table 8:** Clinical and biological characteristics at 1 month post-FMT. Comparative analysis between the study group and the control group.

Variable	Study Group (*n* = 6)	Control Group (*n* = 13)	*p*-Value
BMI (kg/m^2^)	23.2 (22.4–28.7)	26.7 (24.9–29.4)	0.430
Serum Amylase (U/L)	86 (86–89)	62 (49–90)	0.792
Total Bilirubin (mg/dL)	1.4 (1.4–2.8)	1.1 (0.6–1.4)	0.134
Creatinine (mg/dL)	0.7 (0.6–0.8)	0.8 (0.6–0.9)	0.260
Blood Glucose (mg/dL)	111 (99–115)	100 (90–116)	0.455
Sodium (mmol/L)	136 (136–137)	138.5 (137–139)	**0.029**
Potassium (mmol/L)	4.5 (4.1–4.7)	4.4 (3.7–4.9)	0.888
Chloride (mmol/L)	102 (101–103)	104.5 (99–106.9)	0.574
C-Reactive Protein (CRP) (mg/L)	2.3 (1.6–2.8)	7.7 (3–15.1)	**0.034**
TGO (U/L)	39 (31–39)	39 (31–73)	0.792
TGP (U/L)	30 (20–31)	24 (21–30)	0.629
Urea (mg/dL)	26 (21–28)	27 (17–35)	0.539
Prothrombin Time (PT) (seconds)	13.5 (13–14.7)	14.3 (12.2–14.8)	0.792
INR	1.2 (1.1–1.3)	1.2 (1.1–1.3)	0.629
APTT (seconds)	32.1 (30.2–35.7)	38.9 (30.7–43)	0.459
APTT Ratio	1.1 (1.0–1.2)	1.3 (1.0–1.4)	0.459
Leukocytes (cells/μL)	7.4 (6.7–7.4)	8.6 (7.2–9.9)	0.136
Erythrocytes (cells/μL)	4.6 (4.5–4.7)	4.2 (3.7–4.6)	0.792
Hemoglobin (g/dL)	14.8 (13.6–15)	13 (10.6–14.5)	0.334
Hematocrit (%)	41.5 (40.8–43.1)	36.9 (32.8–41.5)	0.510
MCV (Mean Corpuscular Volume) (fL)	92 (91.1–94.3)	90.2 (86.4–94.2)	0.313
Platelets (cells/μL)	121 (120–166)	164.5 (130–255)	0.661
Neutrophils (cells/μL)	3.7 (3.6–4.6)	5.4 (4–6.5)	0.293
Lymphocytes (cells/μL)	1.9 (1.7–1.9)	2.2 (1.9–2.5)	0.219
Monocytes (cells/μL)	0.7 (0.7–0.8)	0.8 (0.7–1.1)	0.236
Basophils (cells/μL)	0.06 (0.05–0.06)	0.06 (0.05–0.08)	0.188
Eosinophils (cells/μL)	0.5 (0.2–0.6)	0.2 (0.1–0.3)	0.136
Neutrophil-to-Lymphocyte Ratio (NLR)	2.3 (1.9–2.4)	2.5 (1.6–5.1)	0.380
Hepatic Encephalopathy			
Grade 0	5 (83.3%)	3 (23.1%)	**0.043**
Grade 1	1 (16.6%)	7 (53.8%)	
Grade 2	0 (0%)	3 (23.1%)	
Child–Pugh Classification			
Child–Pugh Class A	3 (50%)	2 (16.7%)	0.078
Child–Pugh Class B	2 (33.3%)	10 (83.3%)	
Child–Pugh Class C	1 (16.7%)	0 (0%)	
FIB-4 Index	3.3 (2.3–3.8)	3.1 (1.5–5.0)	0.832
APRI Score	0.9 (0.7–1.0)	0.9 (0.6–1.3)	0.898
Liver Elasticity (kPa)	22.6 (20.5–23.6)	23 (22.2–24.9)	0.244
Controlled Attenuation Parameter (CAP) (dB/m)	157.5 (155–186)	170.5 (155–218)	0.213

## Data Availability

The data presented in this study are available on request from the corresponding author due to privacy, legal and ethical reasons.
